# Antiproliferative, Antibacterial and Antifungal Activity of the Lichen *Xanthoria parietina* and Its Secondary Metabolite Parietin

**DOI:** 10.3390/ijms16047861

**Published:** 2015-04-09

**Authors:** Adriana Basile, Daniela Rigano, Stefano Loppi, Annalisa Di Santi, Angela Nebbioso, Sergio Sorbo, Barbara Conte, Luca Paoli, Francesca De Ruberto, Anna Maria Molinari, Lucia Altucci, Paola Bontempo

**Affiliations:** 1 Department of Biological Sciences—Plant Biology Section, University of Naples “Federico II”, Naples 80126, Italy; E-Mails: adbasile@unina.it (A.B.); bbconte@yahoo.it (B.C.); francesca.deruberto@gmail.com (F.D.R.); 2 Department of Pharmacy, University of Naples “Federico II”, Naples 80131, Italy; E-Mail: drigano@unina.it; 3 Department of Life Sciences, University of Siena, Siena 53100, Italy; E-Mails: stefano.loppi@unisi.it (S.L.); paoli4@unisi.it (L.P.); 4 Department of Biochemistry, Biophysics and General Pathology, Second University of Naples, Naples 80138, Italy; E-Mails: annalisa.disanti@unina.it (A.D.S.); angela.nebbioso@unina2.it (A.N.); annamaria.molinari@unina2.it (A.M.M.); 5 Interdepartmental Service Centre for Electron Microscopy C.I.S.M.E., University of Naples “Federico II”, via Foria 223, Naples 80139, Italy; E-Mail: sergio.sorbo@unina.it; 6 Institute of Genetics and Biophysics (IGB), Adriano Buzzati Traverso, Naples 80131, Italy

**Keywords:** *Xanthoria parietina*, lichens, parietin, antimicrobial activity, anticancer activity, signal transduction, proliferation, differentiation

## Abstract

Lichens are valuable natural resources used for centuries throughout the world as medicine, food, fodder, perfume, spices and dyes, as well as for other miscellaneous purposes. This study investigates the antiproliferative, antibacterial and antifungal activity of the acetone extract of the lichen *Xanthoria parietina* (Linnaeus) Theodor Fries and its major secondary metabolite, parietin. The extract and parietin were tested for antimicrobial activity against nine American Type Culture Collection standard and clinically isolated bacterial strains, and three fungal strains. Both showed strong antibacterial activity against all bacterial strains and matched clinical isolates, particularly against *Staphylococcus aureus* from standard and clinical sources. Among the fungi tested, *Rhizoctonia solani* was the most sensitive. The antiproliferative effects of the extract and parietin were also investigated in human breast cancer cells. The extract inhibited proliferation and induced apoptosis, both effects being accompanied by modulation of expression of cell cycle regulating genes such as p16, p27, cyclin D1 and cyclin A. It also mediated apoptosis by activating extrinsic and intrinsic cell death pathways, modulating Tumor Necrosis Factor-related apoptosis-inducing ligand (TRAIL) and B-cell lymphoma 2 (Bcl-2), and inducing Bcl-2-associated agonist of cell death (BAD) phosphorylation. Our results indicate that *Xanthoria parietina* is a major potential source of antimicrobial and anticancer substances.

## 1. Introduction

Lichens are symbiotic organisms in which fungi and algae and/or cyanobacteria form an intimate biological union [1]. They are commonly found worldwide and can survive a variety of harsh environmental conditions. The worldwide lichen flora is estimated to include about 18,500 species that inhabit virtually all terrestrial ecosystems, from the arctic tundra to desert climates and from the lowlands to the highest mountains [1]. Lichens have long had a number of practical applications, including their use as a source of medicinal substances. Their flexibility in habitat occupancy enables them to produce numerous unique secondary metabolites that can comprise up to 30% of the dry weight of a lichen thallus, although 5%–10% is more common. The physiological cost in energy and carbon used to produce these compounds suggests that they play an important role in protection and defense mechanism(s); essentially, lichens produce protective secondary metabolites that serve to deter herbivory and colonization by pathogens [2]. To date, over 800 secondary metabolites of lichens have been discovered, most of which are exclusively present in lichens, and many of which exert a wide variety of biological actions including antibiotic, antimicrobial, antiviral, anti-inflammatory, analgesic, antipyretic, antiproliferative and cytotoxic effects [3,4,5]. Lichens have been used in the traditional medicines of Native Americans and Chinese, principally as expectorants, to treat a range of ailments. During the Middle Ages, lichens were widely used in the herbal remedies of practitioners. Accounts appear in herbal medicine texts of several lichens thought to have therapeutic activity, including species belonging to the genera *Cladonia*, *Evernia*, *Lobaria*, *Parmelia*, *Peltigera*, *Pertusaria*, *Physcia*, *Roccella*, *Usnea* and *Xanthoria* [6]. Specifically, the orange-yellowish species *Xanthoria parietina* (Linnaeus.) Theodor Fries has been listed in various pharmacopoeias [7]. This foliose lichen has a worldwide distribution, being present in Australia, Africa, Asia, North America and Europe. It can be found on rocks, walls (hence the epithet *parietina* meaning “on walls”), roofs, the bark of trees and shrubs, and a wide range of anthropogenic substrata [7]. Because of its color, this lichen has been used against jaundice in traditional medicine since antiquity. In eastern Andalucia (Spain) *Xanthoria parietina* was used to treat menstrual complaints, kidney disorders and as an analgesic [4]. The particular coloring of *Xanthoria parietina* is due to its main secondary metabolite, parietin, an anthraquinone compound with an orange-yellowish color that absorbs blue light. Parietin is synthesized by the mycobiont, and protects the photobiont against oxidation by excessive solar radiation [8]. Among all the secondary metabolites reported for lichens, only a relatively small number (50–60) occur in non-lichenized fungi or higher plants. Parietin is one of these, being present in other fungi such as *Aspergillus* and *Penicillium*, as well as in the vascular plant genera *Rheum*, *Rumex* and *Ventilago* [3].

While the numerous activities of lichen metabolites are now recognized, their therapeutic potential has not been fully explored and remains pharmaceutically unexploited [9]. In particular, although lichens are a rich source of biologically active compounds and more than one thousand secondary metabolites have been identified, very few have been tested for their biological effects. For instance, a relatively small number (~50 species) have been screened for antibiotic activity, despite the fact that over 50% of the lichens tested show at least some antibiotic action [4]. Therefore, research needs to be expanded in this area of study, with further efforts directed towards screening the biological properties of lichen extracts and specific lichen secondary metabolites in order to identify the precise mechanisms of action of active extracts and compounds. The aim of this study was to investigate the antibacterial and antifungal activity of *Xanthoria parietina* and its main secondary metabolite parietin, as well as their antiproliferative and anticancer properties in cancer cell lines.

## 2. Results and Discussion

### 2.1. Antimicrobial Activity of Acetone Extract from Xanthoria parietina and Parietin

Acetone extract (AE) from *Xanthoria parietina* and parietin were analyzed for their antimicrobial activity against nine bacterial strains selected as representative of the Gram (+) and Gram (−) classes, and against clinical isolates of the same strains, all pathogens for humans and known to cause respiratory, gastrointestinal, skin and urinary disorders. The results are shown in Table 1. Minimal inhibitory concentration (MIC) and minimum bactericidal concentration (MBC) values indicate that AE exerted a strong antibacterial activity against the nine American Type Culture Collection (ATCC) bacterial strains and the matched clinically isolated (CI) strains. Specifically, AE inhibited all the tested microorganisms at concentrations of 7.8–62.5 µg/mL. Parietin likewise displayed a robust antibacterial activity, with MICs ranging from 7.8 to 62.5 µg/mL. The Gram (+) *Staphylococcus aureus* from both standard and clinical sources showed the highest sensitivity to both AE and parietin (MIC = 7.8 µg/mL for standard strains and MIC = 15.6 µg/mL for clinical isolates), and also showed interesting MBC values (62.5 µg/mL for standard strains). Generally, among all the bacteria tested, the Gram (+) strains were those most strongly inhibited by AE and parietin. Both treatments demonstrated a significant activity against Gram (−) bacteria, particularly against *P. vulgaris* and *P. mirabilis* (MIC = 15.6 µg/mL for both). Among the others, *S. typhi* and *E. aerogenes* were particularly sensitive to AE and parietin, respectively. Interestingly, *P. mirabilis*, *S. typhi* and *E. cloacae* were the only Gram (−) bacteria for which we also observed interesting MBC values within the tested concentrations.

**Table 1:** Minimal inhibitory concentration (MIC) and minimum bactericidal concentration (MBC) values (μg/mL) of acetone extract (AE) from *Xanthoria parietina* and parietin, and MICs of reference antibiotics.

Microrganism	MIC					MBC	
	AE	Parietin	CTAX	PENG	TET	AE	Parietin
*S. aureus* ATCC 13709	7.8 ± 0.1	7.8 ± 0.2	2 ± 0.1	0.03 ± 0	2 ± 0.1	62.5 ± 0.6	62.5 ± 0.8
*S. aureus* CI	15.6 ± 0.3	15.6 ± 0.4	R	R	R	>100	>100
*E. faecalis* ATCC 14428	15.6 ± 0.1	7.8 ± 0.3	R	8 ± 0.2	2 ± 0.1	>100	62.5 ± 0.7
*E. faecalis* CI	31.3 ± 0.2	15.6 ± 0.1	R	R	R	>100	>100
*P. vulgaris* ATCC 12454	15.6 ± 0.3	15.6 ± 0.2	2 ± 0.1	4 ± 0.3	R	R	R
*P. vulgaris* CI	31.3 ± 0.1	31.3 ± 0.3	32 ± 0.3	R	R	R	R
*P. mirabilis* ATCC 7002	15.6 ± 0.1	15.6 ± 0.1	0.03 ± 0	4 ± 0.2	32 ± 0.6	62.5 ± 0.7	62.5 ± 0.4
*P. mirabilis* CI	15.6 ± 0.1	31.3 ± 0.2	32 ± 0.6	R	R	R	R
*S. typhi* ATCC 19430	15.6 ± 0.2	31.3 ± 0.4	0.5 ± 0.1	4 ± 0.2	1 ± 0.3	62.5 ± 0.5	>100
*S. typhi* CI	31.3 ± 0.2	62.5 ± 0.2	1 ± 0.1	2 ± 0.1	1 ± 0.1	>100	>100
*E. cloacae* ATCC 10699	31.3 ± 0.0	31.3 ± 0.3	R	4 ± 0.4	R	>100	>100
*E. cloacae* CI	62.5 ± 0.1	62.5 ± 0.2	R	R	R	>100	>100
*E. aerogenes* ATCC 13048	31.3 ± 0.2	15.6 ± 0.1	R	4 ± 0.1	R	R	R
*E. aerogenes* CI	62.5 ± 0.5	31.3 ± 0.2	R	R	R	R	R
*P. aeruginosa* ATCC 27853	31.3 ± 0.1	31.3 ± 0.3	16 ± 0.3	R	32 ± 0.1	R	R
*P. aeruginosa* CI	62.5 ± 0.2	62.5 ± 0.1	32 ± 0.4	R	R	R	R
*K. pneumoniae* ATCC 27736	62.5 ± 0.3	31.3 ± 0.2	0.1 ± 0.0	R	16 ± 0.1	R	R
*K. pneumoniae* CI	>100	62.5 ± 0.7	32 ± 0.4	R	R	R	R

CTAX = Cefotaxime; PENG = Benzyl Penicillin Sodium; TET = Tetracycline; CI = Clinically Isolated; R = Resistant. The values shown represent the average of three determinations ± standard deviations.

### 2.2. Antifungal Activity of Acetone Extract from Xanthoria parietina and Parietin

The activity of AE and parietin against the fungal microorganisms tested was also considerable (Table 2), with the highest values for *Rhyzoctonia solani* (MIC = 50 µg/mL for AE and MIC = 31.3 µg/mL for parietin). Parietin was also active against *Botrytis cinerea* and the clinical isolate of *Candida albicans* (MIC = 62.5 µg/mL for both), while the extract was inactive against these two fungi. Therefore, while AE and parietin showed a similar activity on the nine bacterial strains, parietin was more active than AE on the three fungi tested.

**Table 2:** Antifungal activity (MIC values µg/mL) of acetone extract (AE) from *Xanthoria parietina* and parietin.

Microrganism	MIC			MFC	
	AE	Parietin	KCON	AE	Parietin
*Rhyzoctonia solani*	62.5 ± 0.3	31.3 ± 0.5	0.2 ± 0.0	–	–
*Botridis cinerea*	>100	62.5 ± 0.4	0.2 ± 0.1	–	–
*Candida albicans* CI	>100	62.5 ± 0.7	0.4 ± 0.1	–	–

KCON = Ketoconazole; CI = Clinically Isolated; – not active; MFC = Minimum Fungicidal Concentration. The values shown represent the average of three determinations ± standard deviations.

### 2.3. Anticancer Action of Acetone Extract from Xanthoria parietina and Parietin

**Figure 1:**
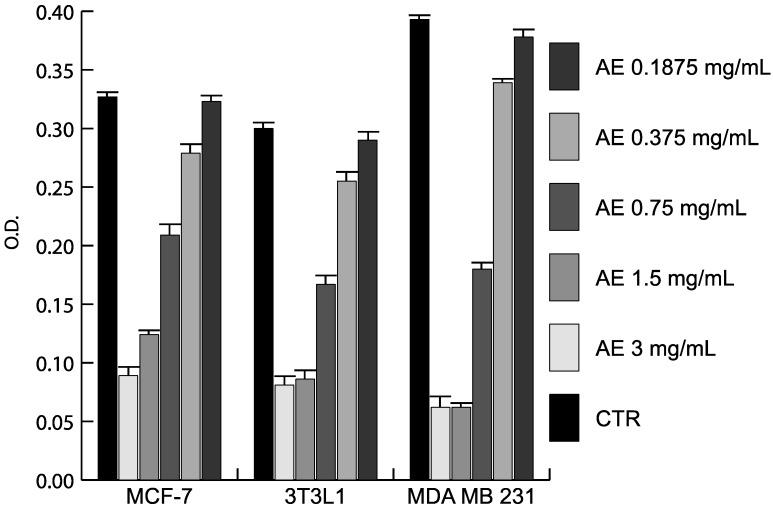
Antiproliferative action of the *Xanthoria parietina* extract. Proliferation curves by crystal violet assay after 3 days of treatment at different doses in the indicated cell lines. Results are the average of experiments performed in triplicate.

**Figure 2:**
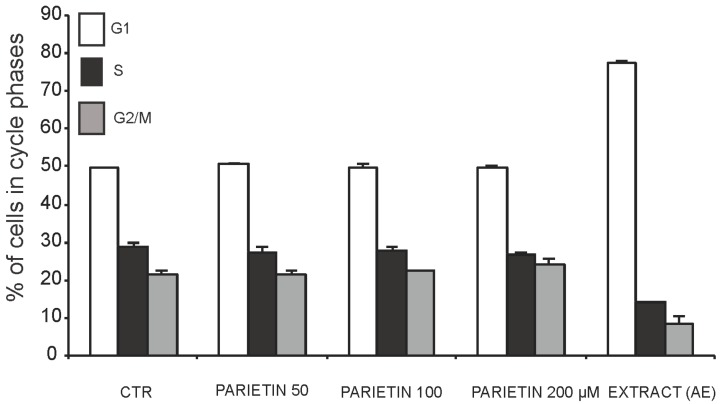
Cell cycle analysis in MDA-MB231 cells at 48 h after treatment with the *Xanthoria parietina* extract (1.5 mg/mL) or parietin at the indicated concentrations.

**Figure 3:**
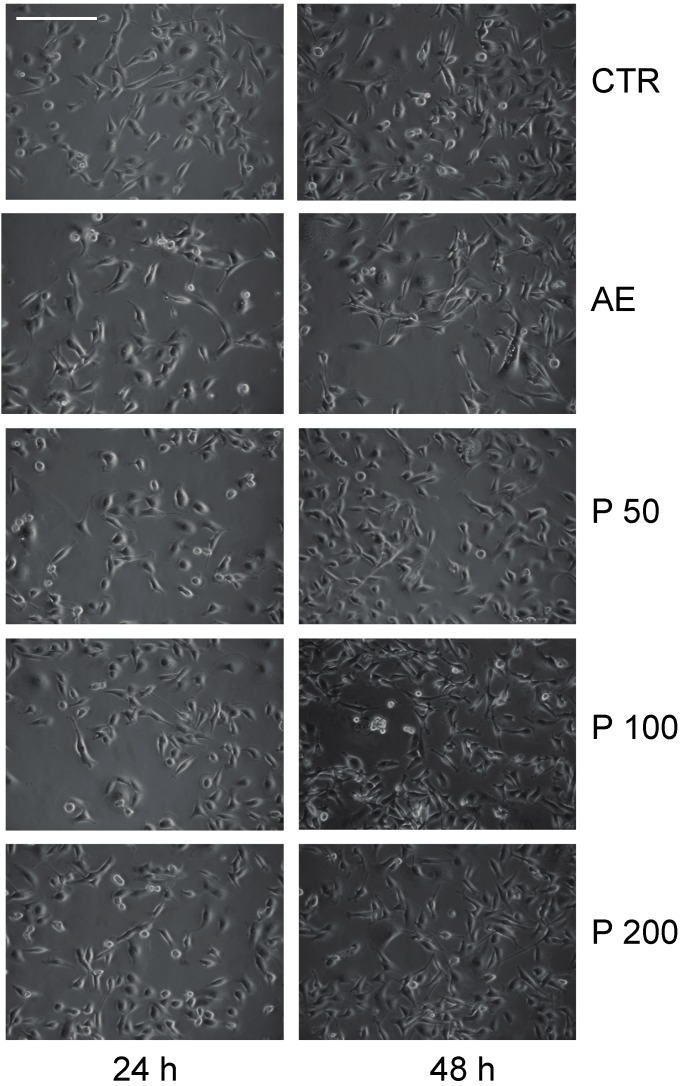
Morphological analysis of proliferation arrest in MDA-MB231 breast cancer cells at 24 and 48 h after treatment with 1.5 mg/mL of the *Xanthoria parietina* extract (AE) or parietin (P) at the indicated concentrations (50-100-200 µM). Control (CTR), scale bar: 71 μm.

The antiproliferative and anticancer properties of different doses of AE and parietin was assessed in MCF-7 and MDA-MB231 breast cancer cells, and in 3T3L1 cells (Figure 1). Using crystal violet staining, proliferation curve analysis revealed the antiproliferative activity of AE in two cancer cell lines tested. As shown in Figure 1, the antiproliferative effect was dose-dependent, with the greatest effect being observed upon three days of treatment using 1.5–3 mg/mL AE. To further investigate the anticancer action of *Xanthoria parietina* extract, we tested its biological effects on the cell cycle in MDA-MB231 cancer cells, the more susceptible of the two lines to treatment. As shown in Figure 2, cells were blocked in G1 phase after 48 h of treatment with 1.5 mg/mL AE. In contrast, parietin alone did not have any effect on cell cycle phases (Figure 2), even when doses were increased. These data were also supported by morphological analyses (Figure 3).

**Figure 4:**
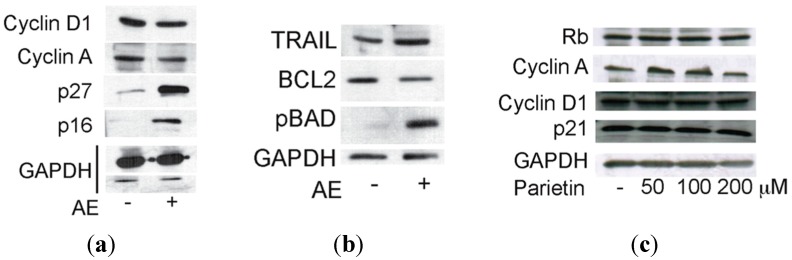
Western blot analysis of molecular effects of *Xanthoria parietina* extract (AE) and parietin in breast cancer cells after 48 h of treatment at the indicated concentrations. (**a**) Molecular effects of AE on cell cycle markers; (**b**) Molecular effects of AE on apoptotic markers; (**c**) Molecular effects of parietin on cell cycle markers. GAPDH expression levels indicate equal loading.

To understand which molecular events underlie the effects of the full extract, we tested its action on known cell cycle regulators after 48 h of treatment with 1.5 mg/mL. As shown in Figure 4A, p27 and p16 were up-regulated, whereas the well-known regulators of proliferation cyclin D1 and cyclin A were decreased. This scenario is reminiscent of a cell cycle block regulated at molecular level. In addition, we found that TNF-related apoptosis-inducing ligand (TRAIL), a key apoptotic player, is already up-regulated at day 2 of treatment. At mitochondria cell death level, B-cell lymphoma 2 (Bcl-2) was decreased together with increased phosphorylation of Bcl-2-associated agonist of cell death (BAD) (Figure 4B), thus indicating that both pathways (extrinsic and intrinsic) are activated. In full agreement with the cell cycle data and morphological analyses, parietin failed to modulate these targets even when doses were increased (Figure 4C), suggesting that multifactorial components of AE contribute to its anticancer action.

### 2.4. Discussion

In the present study, we investigated the antimicrobial, antifungal and antiproliferative activities of AE from the lichen *Xanthoria parietina* and its secondary metabolite parietin. For our research, we used an AE of the lichen, as it has been demonstrated that typical secondary metabolites of lichens are more soluble in acetone than in methanol or other solvents [10].

Overall, our results on the antimicrobial properties of the lichen indicated that the antibacterial activity of AE and parietin is stronger than their antibacterial effect. This finding is consistent with previous studies on the antimicrobial action of lichens [11], which showed that bacteria are more sensitive to antimicrobial activity than fungi due to differences in cell wall composition and permeability. The cell wall of fungi is in fact poorly permeable and consists of polysaccharides such as hitchin and glucan [11]. After all, it makes biological sense that fungal metabolites show a low toxicity to fungi. In terms of antibacterial activity, although parietin was more active than the extract against some bacterial strains, their activity was generally comparable. Both AE and the purified parietin exerted a strong inhibition above all against Gram (+) but also Gram (−) bacteria, unlike the majority of compounds from lichens, which generally inhibit mainly the growth of Gram (+) bacteria [1]. Noteworthy, antibiotic-resistant Gram (−) bacteria, such as *Salmonella* and *Proteus*, were also inhibited. In addition, we observed a significant activity against *Pseudomonas aeruginosa*, which is responsible for severe opportunistic infections and is very often resistant to conventional antibiotics. Furthermore, comparing the activities against ATCC and CI strains, we found similar effects, although almost all the CI bacteria were resistant to more than one reference antibiotic. Interestingly, bacteria isolated from antibiotic-resistant patients proved to be highly sensitive to both AE and parietin. Their activity against the fungal microorganisms tested was also considerable. Our findings are consistent with previous studies investigating the activity of parietin against different bacterial strains and fungi [12,13,14]. However, this is the first time that the antimicrobial and antifungal properties of the extract of *Xanthoria parietina* have been demonstrated.

Antibiotic properties of lichens are of special interest to scientists and their biological role has been relatively well studied [15]. Some lichen compounds inhibit bacterial growth at much lower concentrations compared to other sources of antibiotic therapies. Moreover, they have been shown to be potent not only against sensitive strains of bacteria, but also against various multi-drug resistant bacterial strains. Usnic acid, a phenolic secondary metabolite in lichens, is currently employed as a wide-spectrum antibiotic: its sodium salt is used for treating wounds, burns and fissures [16]. Both the (+) and (−) enantiomers of usnic acid are effective against a large variety of bacterial strains, including strains from clinical isolates. Of particular significance is the inhibition of the growth of multiresistant strains of *Streptococcus aureus*, *enterococci* and mycobacteria. Usnic acid, together with evernic acid and vulpinic acid, inhibits the growth of the resistant *Staphylococcus aureus*, *Bacillus subtilis* and *Bacillus megaterium* [1], and, interestingly, usnic acid and atranorin showed antimicrobial and antibiofilm activity against methicillin-resistant *Staphylococcus aureus* from cystic fibrosis patients [17]. Another interesting antimicrobial compound from lichens is pannarin, which has been shown to be active against multi-drug methicillin-resistant *Staphylococcus aureus* clinical isolates [18]. In terms of antifungal activity, extracts of different lichens such as *Protousnea poeppigii* and *Usnea florida* exhibited antimicrobial activity against different pathogenic fungi, with isodivaricatic acid, 5-propylresorcinol, divaricatinic acid and usnic acid identified as antifungal agents [19], while the lichen *Parmelia reticulate*, along with its metabolites protolichesterinic acid and atranorin, were active against soilborne pathogenic fungi [20]. Our results therefore confirm the importance of lichens as a source of antimicrobial metabolites.

We also assessed the efficacy of different doses of *Xanthoria parietina* extract and parietin in breast cancer cell lines. Lichen secondary products began to be used as anti-cancer drugs in the late 1960s when the activity of lichen polysaccharides against tumor cells was initially explored; similarly, Kupchan and Kopperman [21] first reported the tumor inhibitor activity of usnic acid extracted from *Cladonia* sp. against Lewis lung carcinoma. Since these early studies, many other lichen compounds, either in crude extract or purified form, have been screened against various malignant cell lines, often demonstrating significant inhibitory activity even at very low concentrations [5]. Some of the most promising metabolites are usnic acid, found to be effective against Lewis lung carcinoma, murine P388 leukemia and many other forms of cancer, pannarin, found to inhibit cell growth and to induce cell death in human prostate carcinoma DU-145 cells [22] and human melanoma cells [23], and atranorin, an activator of programmed cell death in A2780 and HT-29 cell lines, probably through the mitochondrial pathway [10,24]. Various lichen metabolites have been found to block cancer cell growth at the sub-G1 or *S*-phase of the cell cycle. The mechanism of cell death in various cancer cell lines induced by these compounds includes apoptosis [22,23,24], both caspase dependent and caspase independent [23]. However, until now there are no studies documenting caspase activation though the death receptor pathway, although there is evidence of mitochondria-mediated caspase activation [5]. We therefore investigated the antiproliferative effects of both *Xanthoria* *parietina* extract and parietin on other cell lines, examining the specific mechanism of action against cancer cells. Previously, Triggiani *et al.* [25] showed that *Xanthoria parietina* extract exerts antiproliferative activity on murine myeloma cells (P3X63Ag8.653) and significantly reduces cell proliferation by up to 75%. In two different studies a slight sensitivity of nine human cancer cell lines to the antiproliferative/cytotoxic effect of parietin was also described [10,24]. Here, we extend the investigation on the antiproliferative effects of both *Xanthoria parietina* extract and parietin on other mammalian cell lines, examining also the specific mechanism of action against cancer cell lines.

*Xanthoria parietina* AE showed a dose-dependent antiproliferative activity in MCF-7 and MDA-MB231 cells, with the greatest effect being obtained using 1.5–3 mg/mL of the extract. To investigate the anticancer potential of AE, we tested its biological effects on cell cycle and apoptosis. The cells were blocked in G1 phase after 48 h of treatment with 1.5 mg/mL of the extract. Interestingly, we demonstrated the presence of cell cycle block and death, occurring with *Xanthoria parietina* treatment, with induction of p27 and p16, whereas cyclin D1 and cyclin A, known to modulate proliferation, were decreased. Notably, under similar experimental conditions, the expression level analysis of pro- and anti-apoptotic proteins showed that Bcl-2 was decreased, while TRAIL and pBAD were induced after treatment with the extract, thus validating its pro-apoptotic action. These lines of evidence strongly suggest the involvement of death receptors and mitochondrial-mediated death pathways in the regulation of apoptosis induced by the extract. In full agreement with cell cycle progression effects and morphological characterizations, parietin alone failed to modulate these targets even when doses were increased. Indeed, previous papers [10,24] showed that other bioactive secondary metabolites isolated from lichens (such as usnic acid and atranorin) were more effective anticancer compounds than parietin. The fact that treatment with parietin alone did not induce any antiproliferative effect clearly indicates that the biological activity of the extract cannot be correlated to parietin alone, but it is probably a result of the synergic action of different components present in the lichen thallus, which together contribute to the overall anticancer effect. Our results highlight the need for continued efforts in screening to explore putative anticancer properties and the pharmaceutical potential of both lichen extracts and specific lichen metabolites using different cell lines. Further studies will be necessary to identify the compounds that are responsible for the observed antitumour effect of the total *Xanthoria parietina* AE.

## 3. Experimental Section

### 3.1. Plant Material and Extraction Procedures

Samples of *Xanthoria parietina* were collected in September 2011 at a remote locality in Tuscany (Pian del Lago, Siena: 11°15'05''E, 43°20'20''N, 230 m a.s.l.), from sun-exposed *Prunus* shrubs. The voucher specimen of the lichen (Voucher No. 187) was deposited at the Department of Biological Sciences, University of Naples “Federico II”, Naples, Italy.

After collection, the samples were brought to the laboratory within 1 h, air-dried at room temperature and at low light intensity for 3 days and subsequently stored at −20 °C until use, according to the recommended method of long-term storage of lichens for later studies [26].

The whole sample was then blended and then extracted over 3 days with 100% acetone at room temperature (3 × 5 L). Following filtration, the solvent was evaporated under reduced pressure and moderate temperature (35 °C) to give a gum (48 g) that was then filtered through a 0.45 µm filter. One part was successively assayed for its biological activity, while another portion was dissolved in CHCl_3_ and then subjected to silica gel column chromatography (30 × 6 cm; silica gel 60 Merck 70–230 mesh, deactivated with 15% H_2_O), eluting with *n*-hexane/EtOAc (from 100:0 to 0:100 gradient), to afford 40 fractions of 200 mL each. The fractions, analyzed by Thyn-Layer Chromatography (eluent system petrol/EtOAc (1:1, *v*/*v*), spray reagent Ce(SO_4_)_2_ in H_2_SO_4_), were opportunely gathered giving 5 fractions (A–E). Fraction C, which was found to be the most active, was further purified by normal-phase semi-preparative HPLC (high-performance liquid chromatography) as described by Dias and Urban [27] to yield parietin (0.05% dry wt.), as resulting by comparison of its spectroscopic data (UV, NMR and MS) with data previously reported [27].

### 3.2. Microorganisms and Media

Nine bacterial strains from the ATTC (Rockville, MD, USA) were employed. They included the Gram-positive (+) bacteria, *Staphylococcus aureus* (ATCC 13709) and *Enterococcus faecalis* (ATCC 14428), and the following Gram-negative (−) bacteria: *Proteus mirabilis* (ATCC 7002), *Proteus vulgaris* (ATCC 12454), *Pseudomonas aeruginosa* (ATCC 27853), *Salmonella typhi* (ATCC 19430), *Enterobacter aerogenes* (ATCC 13048), *Enterobacter cloacae* (ATCC 10699), and *Klebsiella pneumoniae* (ATCC 27736). The same bacterial strains, clinically isolated, were used to compare sensitivity to the extract.

For antimicrobial assays, the extract was added with 5 × 10^−2^ M stock solution in DMSO, and diluted from 0.01 to 1000 µg/mL concentrations in sterile physiological Tris buffer (pH 7.4, 0.05 M) [28] immediately before being used.

### 3.3. Minimal Inhibitory Concentration (MIC) and Minimum Bactericidal Concentration (MBC) Determination

Bacterial strains were grown on MH (Mueller Hinton) agar plates (DIFCO, Detroit, MI, USA) and suspended in MH broth (DIFCO). The MIC values against bacterial strains were determined using the Ericcson and Sherris [29] broth-dilution method (MH broth) as previously described [30]. The inoculum suspensions were prepared from 6-h broth cultures and adjusted to obtain a 0.5 McFarland standard turbidity. The extract was sterilized by filtration through Millipore filters (0.45 µm) (Heidelberg, Germany) and added to MH broth medium. Serial 10-fold dilutions were made for a concentration range between 0.01 and 1000 µg/mL. Two-fold dilutions were tested to obtain a more precise measurement of the MIC. The bacterial suspensions were aerobically incubated for 24 h at 37 °C. The MIC was defined as the lowest concentration able to inhibit any visible bacterial growth. Cultures containing only sterile physiological Tris buffer (pH 7.4, 0.05 M), which did not influence bacterial growth, were used as controls. The MIC values were also determined for tetracycline hydrochloride (Pharmacia, Milan, Italy), benzyl penicillin sodium (Cynamid, Catania, Italy) and cefotaxime sodium (Roussel Pharma, Milan, Italy) in MH broth using standard method.

The MBC determination was carried out by transferring aliquots of bacterial suspensions from the test tubes containing oil concentrations equal or higher (up to 1000 µg/mL) than the MIC to the fresh MH broth. The extract was tested in triplicate and the experiment was run four times.

### 3.4. Antifungal Activity

Antifungal tests were performed against three fungal strains: the CI yeast *Candida albicans*, potentially pathogenic for humans, and the two filamentous phytopathogenic fungi *Botrytis cinerea* and *Rhyzoctonia solani*, kindly provided by the Plant Pathology Department of the University of Naples “Federico II” (Italy).

The fungi were maintained on Sabouraud glucose agar (SGA) medium (Sanofi Diagnostic Pasteur, Chaska, MN, USA).

Two techniques were used for the *in vitro* assays: (1) the agar incorporation method (dilution on a solid medium) and (2) the minimal inhibitory concentration (MIC), which inhibits the visible growth of fungi. Both methods are fully described in a previous report [31]. Positive control cultures were made by culturing the fungi with and without ketoconazole (Sigma, Milan, Italy).

### 3.5. Cancer Cell Lines and Materials

MCF-7 and MDA-MB231 cell lines were obtained from ATCC and routinely cultured. MCF-7 cells were grown at 37 °C in 5% CO_2_ atmosphere in Dulbecco’s Modified Eagle Medium (DMEM, Gibco, New York, NY, USA), supplemented with 5% fetal bovine serum (FBS, Gibco), 1% l-glutamine, 1% ampicillin/streptomycin and 0.1% gentamicin. MDA-MB231 cells, were grown at 37 °C in 5% CO_2_ atmosphere in RPMI-1640 medium (Gibco, NY, USA), supplemented with 10% heat-inactivated FBS, 1% l-glutamine, 1% ampicillin/streptomycin and 0.1% gentamicin. 3T3L1 cells were obtained from ATCC and routinely cultured as previously reported [32].

### 3.6. Crystal Violet Assays

In a 96 multiwell plate (BD), about 1200 cells/well were suspended in 200 µL of cell culture medium. After treatment with extract, cells were fixed in 25 µL of 11% glutaraldehyde in PBS (Sigma) for 15 min, washed in deionized water and dried. Wells were incubated for 20 min in 100 µL of crystal violet (CV; Sigma), 1 mg/mL in 20% methanol, and then washed again. Wells were incubated immediately for 20 min in 100 µL acetic acid (10%). The colorimetric assay carried out in triplicate was quantified by measuring the O.D.595.

### 3.7. Cell Cycle Analysis

Cells (2.5 × 10^5^) were collected and suspended in 500 µL of a hypotonic buffer (0.1% Triton X-100, 0.1% sodium citrate, 50 µg/mL propidium iodide, RNAse A). Cells were incubated in the dark for 30 min. Samples were acquired on a FACS-Calibur flow cytometer and analyzed with standard procedures using Cell Quest software (Becton Dickinson, Milan, Italy) [33] and ModFit LT version 3 software (Verity Software House, Topsham, ME, USA) as previously reported [34,35,36]. All the experiments were run in triplicate.

### 3.8. Western Blot Analyses

Forty micrograms of total protein extracts were separated on a 12% polyacrylamide gel and blotted as previously described [37]. Western blots were performed for p16, p27, cyclin A, cyclin D1 and Bcl-2 (dilution 1:500; Santa Cruz, Santa Cruz, CA, USA). GAPDH (Santa Cruz, dilution 1:500) was used to normalize for equal loading.

To quantify TRAIL, 100 µg total protein extract was separated on 10% polyacrylamide gel and blotted. Western blots were performed on TRAIL (dilution 1:200; Abcam, Cambridge, UK), and GAPDH (dilution 1:1000; Santa Cruz) was used to normalize for equal loading. To determine p-BAD, 40 µg of total protein extract was separated on 12% polyacrylamide gel and blotted. Antibodies used were: anti-BAD, (dilution 1:500; Millipore); GAPDH (dilution 1:500, Santa Cruz), which was used to normalize for equal loading.

### 3.9. Statistical Analysis

One-way analysis of variance and Tukey’s pairwise comparisons (MINITAB Release 11, 1996) were applied to determine the significance of differences in all measured parameters. All data are expressed as means ± standard deviation (SD) of three independent experiments. Statistical significance is identified as follows: *p* < 0.05, *p* < 0.01, *p* < 0.001.

## 4. Conclusions

Results here described show that *Xanthoria parietina* and the metabolites extracted from it could represent a valuable tool in the treatment of cancer and infections, and may contribute to the development of new and safe agents for inclusion in antimicrobial, antifungal and anticancer regimens. However, our findings show that the overall anticancer effect of lichen extract cannot be attributed to its main secondary metabolite parietin, but is probably a result of the synergic action of different components present in the lichen thallus. Hence, further screening of other metabolites from *Xanthoria parietina* lichen will be necessary. With recent advances in technology, the development of cost-effective options for growing and harvesting lichen metabolites commercially as a source of effective drugs against pathogenic bacteria and various forms of cancer is a highly promising avenue to explore.

## Abbreviations

Bcl-2: B Cell Lymphoma 2
BAD: Bcl-2-associated agonist of cell death
HPLC: high-performance liquid chromatography
MBC: Minimum bactericidal concentration
MIC: Minimal inhibitory concentration
MS: mass spectrometry
NMR: nuclear magnetic resonance
TRAIL: TNF-related apoptosis-inducing ligand

## References

[1] Nash T.H. (2008). Lichen Biology.

[2] Dayan F.E., Romagini J.G. (2002). Structural diversity of lichen metabolites and their potential for use. Adv. Microb. Toxin Res. Biotechnol. Explor..

[3] Lawrey J.D. (1986). Biological role of lichen substances. Byrologist.

[4] Boustie J., Grube M. (2005). Lichens—A promising source of bioactive secondary metabolites. Plant Genet. Resour..

[5] Shrestha G., St. Clair L.L. (2013). Lichens: A promising source of antibiotic and anticancer drugs. Phytochem. Rev..

[6] Saklani A., Upreti D.K. (1992). Folk uses of some lichens of Sikkim. J. Ethnopharmacol..

[7] Honegger R., Zippler U., Scherrer S., Dyer P.S. (2004). Genetic diversity in *Xanthoria parietina* (L.) Th. Fr. (lichen-forming ascomycete) from worldwide locations. Lichenologist.

[8] Gauslaa Y., McEvoy M. (2005). Seasonal changes in solar radiation drive acclimation of the sun-screening compound parietin in the lichen *Xanthoria parietina*. Basic Appl. Ecol..

[9] Müller K. (2002). Pharmaceutically relevant metabolites from lichens. Appl. Microbiol. Biot..

[10] Backorova M., Backor M., Mikes J., Jendzelovsky R., Fedorocko P. (2011). Variable responses of different human cancer cells to the lichen compounds parietin, atranorin, usnic acid and gyrophoric acid. Toxicol. In Vitro.

[11] Kosanic M., Manojlovic N., Jankovic S., Stanojkovic T., Rankovic B. (2013). *Evernia. prunastri* and *Pseudoevernia. furfuraceae* lichens and their major metabolites as antioxidant, antimicrobial and anticancer agents. Food Chem. Toxicol..

[12] Manojlovic N.T., Solujic S., Sukdolak S., Krstic L.J. (2000). Isolation and antimicrobial activity of anthraquinones from some species of the lichen genus *Xanthoria*. J. Serb. Chem. Soc..

[13] Manojlovic N.T., Solujic S., Sukdolak S., Milosev M. (2005). Antifungal activity of *Rubia tinctorum*, *Rhamnus frangula* and *Caloplaca cerina*. Fitoterapia.

[14] Tamokou J.D.D., Tala M.F., Wabo H.K., Kuiate J.R., Tane P. (2009). Antimicrobial activities of methanol extract and compounds from stem bark of *Vismia rubescens*. J. Ethnopharmacol..

[15] Crittenden P.D., Porter N. (1991). Lichen-forming fungi: Potential sources of novel metabolites. Trend Biotechnol..

[16] Cocchietto M., Skert N., Nimis P.L., Sava G. (2002). A review on usnic acid, an interesting natural compound. Naturwisseneschaften.

[17] Pompilio A., Pomponio S., di Vincenzo V., Crocetta V., Nicoletti M., Piovano M., Garbarino J.A., di Bonaventura G. (2013). Antimicrobial and antibiofilm activity of secondary metabolites of lichens against methicillin-resistant *Staphylococcus aureus* strains from cystic fibrosis patients. Future Microbiol..

[18] Celenza G., Segatore B., Setacci D., Bellio P., Brisdelli F., Piovano M., Garbarino J.A., Nicoletti M., Perilli M., Amicosante G. (2012). *In vitro* antimicrobial activity of pannarin alone and in combination with antibiotics against methicillin-resistant *Staphylococcus aureus* clinical isolates. Phytomedicine.

[19] Schmeda-Hirschmann G., Tapia A., Lima B., Pertino M., Sortino M., Zacchino S., de Arias A.R., Feresin G.F. (2007). A new antifungal and antiprotozoal depside from the andean lichen *Protousnea poeppigii*. Phytother. Res..

[20] Goel M., Dureja P., Rani A., Uniyal P.L., Laatsch H. (2011). Isolation, characterization and antifungal activity of major constituents of the Himalayan lichen *Parmelia reticulata* Tayl.. J. Agric. Food Chem..

[21] Kupchan S.M., Kopperman H.I. (1975). l-usnic acid: Tumor inhibitor isolated from lichens. Experientia.

[22] Russo A., Piovano M., Lombardo L., Vanella L., Cardile V., Garbarino J. (2006). Pannarin inhibits cell growth and induces cell death in human prostate carcinoma DU-145 cells. Anti-Cancer Drugs.

[23] Russo A., Piovano M., Lombardo L., Garbarino J., Cardile V. (2008). Lichen metabolites prevent UV light and nitric oxide-mediated plasmid DNA damage and induce apoptosis in human melanoma cells. Life Sci..

[24] Backorova M., Jendzelovsky R., Kello M., Backor M., Mikes J., Fedorocko P. (2012). Lichen secondary metabolites are responsible for induction of apoptosis in HT-29 and A2780 human cancer cell lines. Toxicol. In Vitro.

[25] Triggiani D., Ceccarelli D., Tiezzi A., Pisani T., Munzi S., Gaggi C., Loppi S. (2009). Antiproliferative activity of lichen extracts on murine myeloma cells. Biologia.

[26] Honegger R. (2003). The impact of different long-term storage conditions on the viability of lichen-forming ascomycetes and their green algal photobiont, *Trebouxia.* spp.. Plant Biol..

[27] Dias D.A., Urban S. (2009). Phytochemical investigation of the Australian lichens *Ramalina glaucescens* and *Xanthoria parietina*. Nat. Prod. Commun..

[28] Ieven M., Dirk A., Vanden Berghe V., Francis M., Vlietinck A., Lammens E. (1979). Screening of higher plants for biological activities. I. Antimicrobial activity. Planta Med..

[29] Ericcson H.M., Sherris J.C. (1971). Antibiotic sensitivity testing: Report of an international collaborative study. Acta Pathol. Microbiol. Scand..

[30] Bontempo P., Carafa V., Grassi R., Basile A., Tenore G.C., Formisano C., Rigano D., Altucci L. (2013). Antioxidant, antimicrobial and anti-proliferative activities of *Solanum tuberosum* L. var. Vitelotte. Food Chem. Toxicol..

[31] Basile A., Conte B., Rigano D., Senatore F., Sorbo S. (2010). Antibacterial and antifungal properties of acetonic extract of *Feijoa sellowiana* fruits and its effect on *Helicobacter pylori* growth. J. Med. Food.

[32] Nebbioso A., Dell’Aversana C., Bugge A., Sarno R., Valente S., Rotili D., Manzo F., Teti D., Mandrup S., Ciana P. (2010). HDACs class II-selective inhibition alters nuclear receptor-dependent differentiation. J. Mol. Endocrinol..

[33] Miceli M., Franci G., Dell’Aversana C., Ricciardiello F., Petraglia F., Carissimo A., Perone L., Maruotti G.M., Savarese M., Martinelli P. (2013). MePR: A novel human mesenchymal progenitor model with characteristics of pluripotency. Stem Cells Dev..

[34] Nebbioso A., Clarke N., Voltz E., Germain E., Ambrosino C., Bontempo P., Alvarez R., Schiavone E.M., Ferrara F., Bresciani F. (2005). Tumor-selective action of HDAC inhibitors involves TRAIL induction in acute myeloid leukemia cells. Nat. Med..

[35] Nebbioso A., Pereira R., Khanwalkar H., Matarese F., García-Rodríguez J., Miceli M., Logie C., Kedinger V., Ferrara F., Stunnenberg H.G. (2011). Death receptor pathway activation and increase of ROS production by the triple epigenetic inhibitor UVI5008. Mol. Cancer Ther..

[36] Lepore I., Dell’Aversana C., Pilyugin M., Conte M., Nebbioso A., de Bellis F., Tambaro F.P., Izzo T., Garcia-Manero G., Ferrara F. (2013). HDAC inhibitors repress BARD1 isoform expression in acute myeloid leukemia cells via activation of miR-19a and/or b. PLoS ONE.

[37] Franci G., Casalino L., Petraglia F., Miceli M., Menafra R., Radic B., Tarallo V., Vitale M., Scarfò M., Pocsfalvi G. (2013). The class I-specific HDAC inhibitor MS-275 modulates the differentiation potential of mouse embryonic stem cells. Biol. Open.

